# A fatal case of acute Marchiafava-Bignami disease complicated by acute abdomen– a case report

**DOI:** 10.1186/s12245-025-00873-9

**Published:** 2025-04-08

**Authors:** Bence Prohászka, Novák Pál Kaposi, Zsuzsanna Jánosi, Bence Gunda, Ildikó Pákozdy, Szabolcs Gaál-Marschal, Dóra Melicher, Bánk G. Fenyves, Csaba Varga

**Affiliations:** 1 Department of Emergency Medicine, Semmelweis University, Budapest, Hungary; 2 Medical Imaging Center, Semmelweis University, Budapest, Hungary; 3 Department of Neurology, Semmelweis University, Budapest, Hungary

**Keywords:** Chronic alcohol consumption, Ethanol toxicity, Altered mental state, Emergency department

## Abstract

**Background:**

Marchiafava-Bignami Disease (MBD) is a rare disorder characterized by demyelination and necrosis of the corpus callosum, with only 300 documented cases worldwide. Chronic alcohol consumption and vitamin B-complex deficiencies are contributing factors. Acute cases may present with a range of neurological symptoms, including seizures and coma. Subacute and chronic forms can lead to interhemispheric disconnection syndrome and progressive dementia.

**Case presentation:**

We present the case of a young male patient’s first hospital admission due to an acute decline in conscious level. A detailed history revealed regular alcohol consumption and substandard living conditions. The deterioration in consciousness was attributed to the diagnosis of MBD based on neurological signs, characteristic brain imaging findings, and a history of alcohol use. In addition, a small bowel perforation was also diagnosed. Supportive therapy and thiamine were initiated, and the patient was transferred to surgery for an operation. After two surgeries, the perforation was covered. The patient’s level of consciousness showed slight improvement; however, the psychiatrist noted severe cognitive deficits. Ultimately, the patient entered a septic state and passed away.

**Conclusion:**

Acute MBD can potentially cause altered mental state, coma, and death; however, cases can be complicated by other emergency conditions. This case demonstrates the importance of a multidisciplinary approach.

## Background

Marchiafava-Bignami Disease (MBD) was identified in 1903 by Ettore Marchiafava and Amico Bignami during autopsies of patients with alcohol use disorder who died from seizures. They found demyelination and necrosis in the corpus callosum and surrounding white matter. MBD is rare, with only 300 documented cases globally, including a single case from Hungary [1]. Ethanol is the leading risk factor, causing hypovitaminosis, neurotoxicity, oxidative stress, cytotoxic edema, and atrophy [2]. Clinical manifestations can be acute, subacute, or chronic. Acute cases may present with seizures, confusion, altered mental state, and coma. Subacute and chronic forms can lead to interhemispheric disconnection syndrome, with symptoms like ataxia, dysarthria, behavioral issues, hallucinations, and progressive dementia [2, 3]. Patients with suspected MBD should have brain imaging, preferably MRI, to rule out other potential causes of coma or similar symptoms. MBD has two subtypes: subtype A affects the entire corpus callosum, while subtype B affects only partial areas [4]. Several “MBD mimic” conditions can cause a challenge in differential diagnosis. One of the most similar conditions is Wernicke encephalopathy (WE), which is associated with chronic alcohol consumption or malnutrition, with characteristic symptom triad: confusion, ataxia, and oculomotor abnormalities [5]. MBD presents with a variety of symptoms based on the disease stage and affects the corpus callosum, leading to edema and necrosis. In contrast, WE targets areas around the third ventricle, including the medial thalamic nuclei, tectal plate, mammillary bodies, and periaqueductal gray matter [6]. Other neurological diseases like pellagra, meningitis, or encephalitis and conditions affecting the central nervous system, such as liver failure or poisoning, can present with similar symptoms (Table 1). Laboratory tests, including toxicology screenings, help differentiate these conditions from MBD [2]. No specific therapy exists for MBD, so treatment is limited to supportive care. Positive responses have been observed with intravenous thiamine, folate, vitamin B complex, and high-dose corticosteroids. Nutritional support and cessation of alcohol consumption are also recommended [2, 7]. Amantadine has been used in some cases, but its mechanism and benefits are not well established [1, 8]. MBD outcomes vary, with some patients achieving slow, complete recovery while others may deteriorate to terminal illness [2]. This report discusses a case of acute MBD complicated by a life-threatening acute abdomen, emphasizing the need for a multidisciplinary approach.

**Table 1:** Differential diagnostic possibilities

	Marchiafava-Bignami disease (MBD)	Wernicke encephalopathy (WE)	Metabolic enchephalitis	Infective diseases (meningitis, enchephalitis)	Stuctural leasion	Toxication (opioid, carbon-monoxide)
Cause	Alcohol abuse	Alcohol abuse, malnutrition, hyperemesis, malabsorption, inadequate dietary intake, increased metabolic requirement, dialysis patients	Septic state, hepatic (ammonia elevated), uremic (urea elevated), electrocyte abnormalities (natrium, calcium), hypoglycemia, hyperglycemia, hypoxic	Bacterial, viral, fungal infections	Stroke, hemorrhage, traumatic injuries	Opioid overdose, CO toxication
Implication	Thiamin deficiency	Thiamin deficiency	Oedema, neurotransmitter disturbance	Altered blood-brain permeability, inflammatory cytokines	Structural damage of the brain	Opioid overdose, Increased carboxyhemoglobin
Symptoms	*Acute*: seizures, confusion, altered mental state, coma *Subacut and chronic*: interhemispheric disconnection syndrome, ataxia, dysarthria, behavioral issues, hallucination, progressive dementia	Confusion, ataxia, ocular abnormalities	Irritability, apathy, lethargy, confusion, agitation, coma	Diffuse muscle weakness, polyneuropathy, fever, stiff neck, severe headache, nausea, coma	Seizure, motor deficit, sensory deficit, ataxia, dizziness, agnosia, aphasia, dysarthria, headache, homonymous hemianopia, diplopia, nausea, coma	*Opioid*: decreased mental status, respiratory rate, tidal volume, vowel sounds and miotic pupils *CO*: headache, nausea, dizziness, drowsiness, vomiting, caught, confusion, shortness of breath, syncope
Clinical presentation	Variety of symptoms, severe symptoms, improve slowly	Characteristic symptoms, moderate symptoms, resolve within weeks	Confusion, coma with deviation in blood gas or laboratory results. History of vomiting, diarrhoea, bad medicine compliance. Positive meningeal signs	Infection, elevated infection markers, fever with confusion, sudden onset	Gradual and progression or abrubt onset	*Opioid*: Mental state between euphoria and coma *CO*: Fire victims, flu-like symtoms, unexplained altered mental status
Affecting brain areas	*In subtype A*: the whole corpus callosum affected *In subtype B*: partial part of the corpus callosum affected	Medial thalamic nuclei, tectal plate, mammillary bodies, periaqueductal gray matter	Not specific	Not specific	Specific	Not specific
Diagnosis	Laboratory, brain imaging, clinical sympthoms	Laboratory, brain imaging, clinical sympthoms	Blood gas, laboratory	Lumbar punction, Electroenc-ephalography	CT, MRI	*Opioid*: Laboratory *CO*: COHg measurement
Therapy	Thiamine, folate, vitamin B complex, high-dose corticosteroids, amantidine, nutritional support, cessation of alcohol consumption	Thiamine, glucose infusion, nutritional support, cessation of alcohol	Treating the underlying deviation	Infection control	Thrombolysis, neurosurgeon intervention	*Opioid*: Antidotum, supportive therapy *CO*: High-flow oxygen
Outcome	Slow and complete recovery, or terminal illness	Complete recovery, or progression to Korsakoff syndrome	Varying from the cause of the problem, may be reversible	May be reversible	May be reversible	*Opioid*: May be reversible or fatal due respiratory arrest *CO*: Complete recovery or late neurocognitive impairment

### Case presentation

A 40-year-old male was transported by ambulance to the emergency department due to delirium and seizures. He had been found on the floor at home, displaying tremors similar to seizures. Collateral history indicated poor living conditions and declining health over the past month. The patient hadn’t consumed alcohol in two weeks, reported speech difficulties for four days, and had been bedridden for three days. There were no previous hospital visits, and relatives couldn’t provide information on known illnesses or medications.

**Fig. 1:**
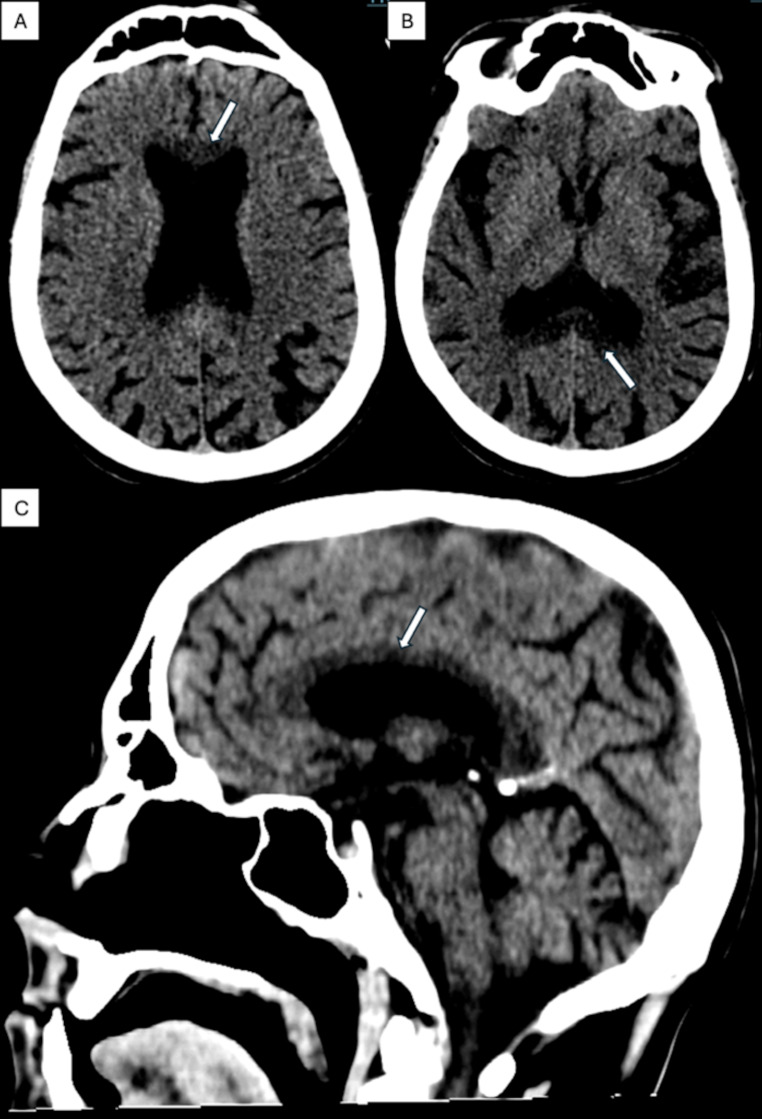
Native head CT images. Panel **A**: Axial CR scan, showing conspicuous hypodensity (arrow) of the genu of the corpus callosum in the area between the frontal horns of the lateral ventricles. Panel **B**: Axial CT scan showing the hypodensity (arrow) of the splenium of the corpus callosum in the area between the occipital horns of the lateral ventricles. The symmetrical narrowing of the gyri and widening of the sulci along the convexity of the hemispheres are in keeping with the diagnosis of generalized brain atrophy. Panel **C**: Sagittal CT scan showing the marked hypodensity (arrow) and thinning of the corpus callosum at the top of the lateral ventricle

**Fig. 2:**
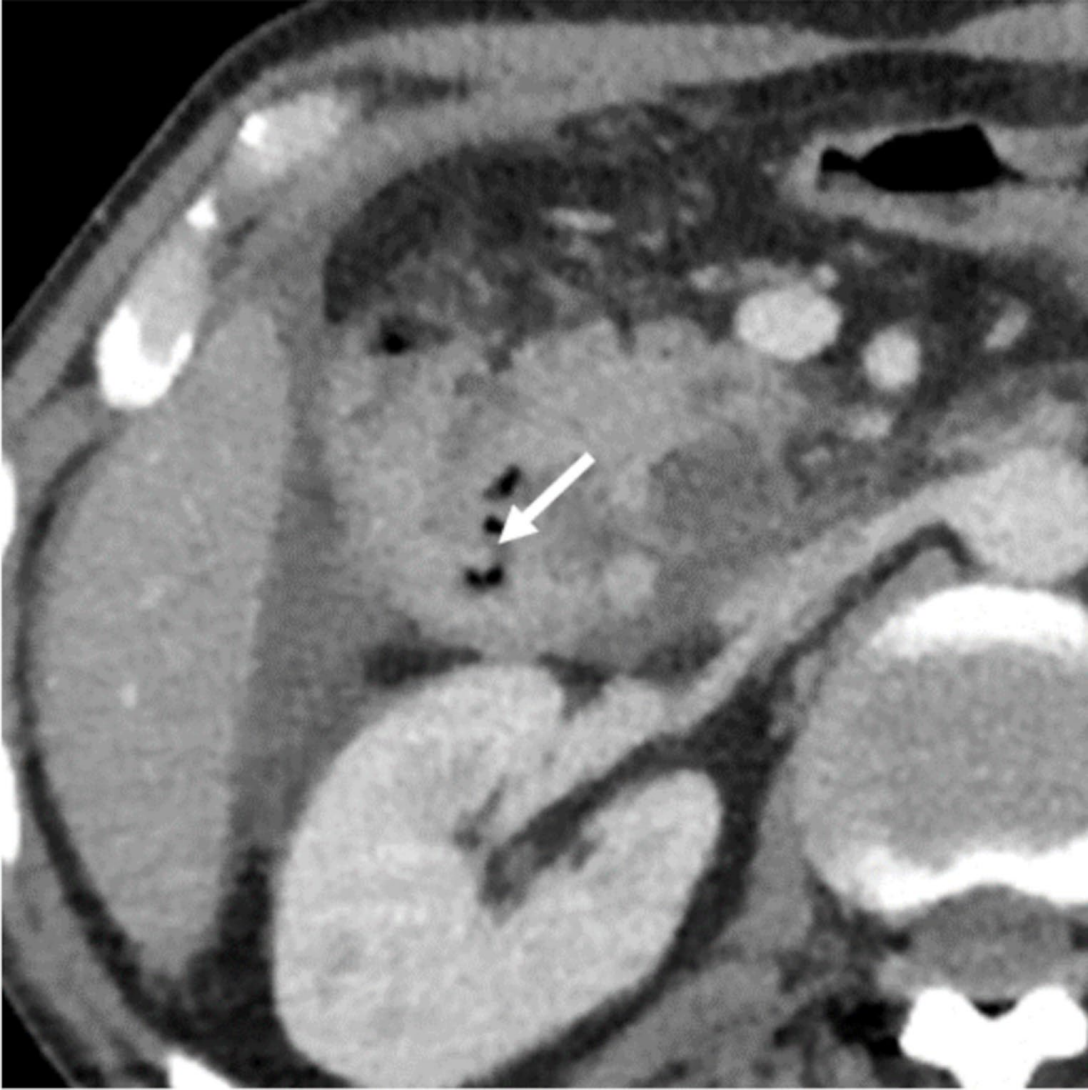
Contrast-enhanced abdominal CT. Wall defect was visible on the descending part of the duodenum (arrow) with extraluminal air and fluid in the pancreaticoduodenal recess and surrounding right liver lobe in keeping with the diagnosis of duodenal perforation

Upon arrival, he was unresponsive and exhibited tachycardia, normal blood pressure, impaired consciousness, and abdominal guarding. There was pain upon abdominal pressure, and a rectal exam showed normochromic stool with a positive fecal occult blood test. Blood gas analysis revealed a hemoglobin level of 103 g/l and a lactate level of 8.5 mmol/l, raising concerns for mesenteric ischemia. Abdominal CT angiography and non-contrast head CT scans were ordered. The emergency head CT revealed severe hypodensity and thinning of the entire corpus callosum, indicative of chronic demyelination and necrosis of the nerve fibers and raising the suspicion of MBD (Fig. 1A-C). In addition, there was a pronounced symmetrical narrowing of the gyri and widening of sulci in both cerebral hemispheres, as well as a widening of the supratentorial ventricular system in keeping with the diagnosis of generalized brain atrophy (Fig. 1B-C). A contrast-enhanced abdominal CT showed signs of duodenal perforation, including extraluminal air and fluid collections and wall defect on the descending part of the duodenum (Fig. 2). Neurology and surgery consultations were ordered promptly. Laboratory tests revealed elevated inflammatory markers, high procalcitonin, macrocytic anemia, increased lipase, and negative ethanol (Table 2). After blood cultures, we continued fluid therapy and thiamine supplementation and initiated broad-spectrum antibiotics. By the time the neurologist arrived, the patient’s consciousness had improved, and he could follow simple instructions. The neurologist confirmed the MBD diagnosis and recommended further thiamine therapy. The patient was admitted to surgery for an operation on day zero.

On day one, the initial surgery revealed diffuse fibrinous peritonitis and a thickened duodenum, but the site of perforation was not found. On day three, the patient was reoperated due to significant discharge from the abdominal drain, revealing and covering a needle-point perforation in the duodenum. During the postoperative days on the surgical ward, consciousness improved, but mental functions were still limited. A psychiatrist consultant noted severe cognitive deficits and dementia and recommended further thiamine therapy. Institutionalization and guardianship were considered. From day five, the patient’s condition deteriorated with bilateral pneumonia, worsening coagulation, pancreatic fluid discharge from the abdominal drain, and significant free abdominal fluid. On day thirteen, cardiac arrest occurred, and the patient was pronounced dead after unsuccessful resuscitation. For technical reasons, the autopsy was only carried out several days after the death. Due to advanced tissue decomposition, the diagnosis of MBD could not be confirmed by the autopsy. According to the pathological report, the cause of death was sepsis caused by a perforated duodenal ulcer and subsequent peritonitis and pancreatitis.

**Table 2:** Laboratory findings

Name (unit)	Value	Reference
C-reactive protein (mg/L)	126	< 10.0
Procalcitonin (ug/L)	20	< 0.50
White blood cells (G/L)	9.93	4.00–10.00
Neutrophil granulocyte (G/L)	8.64	1.80-7.00
Lymphocyte (G/L)	1.09	1.00–4.00
Hemoglobin (g/L)	103	135–170
Hematocrit (L/L)	0.27	0.39–0.52
MCV (fL)	118.5	80.0–99.0
MCH (pg)	22.2	27.0–34.0
MCHC (g/L)	373	315–360
Thrombocyte (G/L)	157	150–400
INR	1.23	
APTI (sec)	34	28.0–40.0
Thrombin time (sec)	26.4	15.0–22.0
Fibrinogen (g/L)	3	1.5-4.0
Glucose (mmol/L)	4.4	4.1–5.9
Urea (mmol/L)	7.9	2.8–7.2
Creatinine (umol/L)	80	59–104
eGFR (ml/min/1.73 m^2^)	> 90	> 90
Total bilirubin (umol/L)	26.4	5.0–21.0
Direct bilirubin (umol/L)	12.4	< 3.4
GOT (U/L)	71	< 50
GPT (U/L)	27	< 50
GGT (U/L)	48	< 50
ALP (U/L)	64	30–120
LDH (U/L)	330	< 248
Lipase (U/L)	199	< 67
Sodium (mmol/L)	135	135–146
Potassium (mmol/L)	3.4	3.5–5.1
Ethyl-alcohol (g/L)	< 0.03	< 1.00

## Discussion

The case presented above was an acute onset MBD: A 40-year-old male patient with a history of alcohol abuse was admitted in an unconscious state, with worsening symptoms over a month. CT scans showed significant hypodensity in the corpus callosum, leading to a radiological diagnosis of type “A” MBD. Continuous thiamine therapy slightly improved the patient’s mental state, but a psychiatrist diagnosed him with severe cognitive disorder and dementia at a young age. The patient likely remained unaware of his symptoms, and poor living conditions prevented him from seeking help, leading to undiagnosed disease. Alcohol use disorder is common, particularly in Hungary, with many cases going unnoticed due to lack of awareness.

MBD can manifest in various forms and mimic conditions such as central nervous system infections (meningitis, encephalitis), metabolic encephalitis, structural lesions, and poisoning from substances like opioids and carbon monoxide, complicating diagnosis (Table 1). These differential diagnostic possibilities could present similar symptoms to MBD, presenting confusion, altered mental status, and behaviour changes.

Central nervous system infectious conditions typically present with fever and elevated inflammatory markers. Although the patient did not exhibit fever, the inflammatory markers were elevated. The neurological examination revealed no meningeal signs, which made infectious causes less likely. Bedside blood gas analysis and laboratory tests are crucial to exclude metabolic encephalopathies. In this case, the initial blood gas analysis did not indicate any of these conditions, and subsequent laboratory tests yielded no significant findings. Neurological conditions caused by structural lesions, such as structural lesions, absent seizure or postictal confusion, can be evaluated based on patient history, circumstances of hospital admission, and neurological examination. Poisoning conditions were excluded because no heteroanamnestic data suggested CO poisoning, and there was no visible carboxyhemoglobin elevation in the blood gas results. Opioid poisoning was also less suspicious because during the patient evaluation, we didn’t find miosis, and the heteroanamnestic information didn’t suggest it either. In this case, these factors did not suggest any of the previously mentioned conditions. Ultimately, given the patient’ s history of alcohol consumption, the presence of severe symptoms, and the absence of metabolic abnormalities, the primary suspected cause of the altered consciousness was a thiamine deficiency- related disorder. Distinguishing between thiamine- related disorders can be challenging, especially in a clinical setting. In our case, the CT imaging results indicated a diagnosis of MBD rather than the more common WE. It is important to note that MBD is always associated with alcohol use disorder, while WE is not exclusively linked to alcohol consumption. MBD typically presents with more severe symptoms, while WE displays milder symptoms with specific characteristic features. In this case, the presence of severe symptoms, clear evidence of alcohol use disorder, and the absence of the characteristic symptoms associated with WE suggest that the latter is less likely, even in a bedside assessment. Ultimately, the CT findings indicative of MBD also showed chronic demyelination, diffuse atrophy of the corpus callosum, and noticeable generalized brain atrophy, underscoring the patient’s poor condition at a relatively young age.

## Conclusion

This case underscores the importance of a comprehensive examination and a multidisciplinary approach. Without a full-body assessment, the abdominal perforation could have been missed. Additionally, it’s important to avoid pre-judging patients, as this could cause practitioners to overlook crucial information that may be significant and lead the workup in another direction. Particular attention should be given to patients with a history of alcohol use disorder who exhibit newly developed behavioral changes, movement abnormalities, signs of dementia, or a seriously impaired mental state, especially in younger patients.

## Data Availability

No datasets were generated or analysed during the current study.

## Abbreviations

ALP: Alkaline phosphatase
APTT: Activated partial thromboplastin time
CT: Computer tomography
eGFR: Estimated glomerular filtration rate
GGT: Gamma-glutamyltransferase
GOT: Glutamic oxaloacetic transaminase
GPT: Glutamic pyruvic transaminase
LDH: Lactate dehydrogenase
MBD: Marchiafava-Bignami disease
MCH: Mean corpuscular hemoglobin
MCHC: Mean corpuscular hemoglobin concentration
MCV: Mean corpuscular volume
MRI: Magnetic resonance imaging
WE: Wernicke encephalopathy
